# Supplementary material for: Reprogramming of bone marrow myeloid progenitor cells in patients with severe coronary artery disease
Source: eLife. 2020 Nov 10;9:e60939. doi: 10.7554/eLife.60939 (PMC7665893; doi:10.7554/eLife.60939)
Supplement: Supplementary file 1. — Monocytes were selected based on CD45+ HLA-DR+ and monocyte scatter properties, after exclusion of dead cells and doublets. Then CD3+ lymphocytes and CD56+ NK-cells were excluded, and monocyte subsets were identified in the CD14/CD16 plot as the percentage of gated. CD11b and CCR2 expression was determined on the monocyte population. [file elife-60939-supp1.pdf]

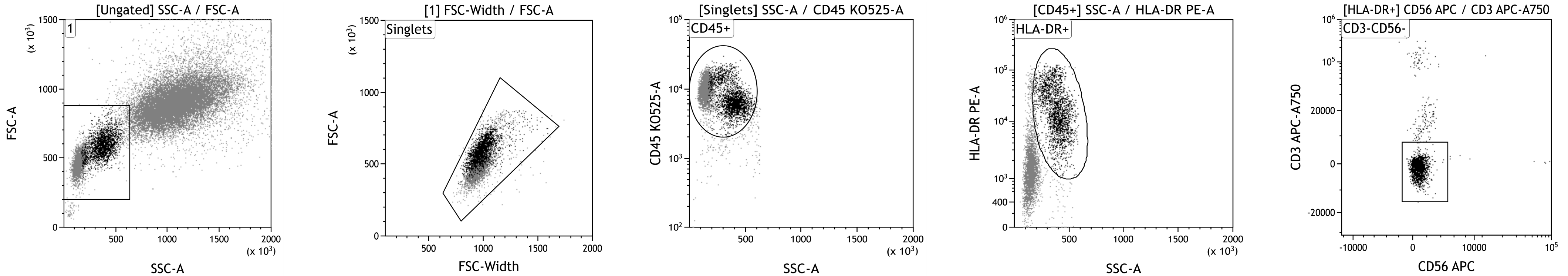

Gate	Number	%Gated
All	26.859	100,00
1	5.614	20,90

Gate	%Gated
All	100,00
Singlets	99,54

Gate	%Gated
All	100,00
CD45+	96,66

Gate	%Gated
All	100,00
HLA-DR+	39,94

Gate	%Gated
All	100,00
CD3-CD56-	91,32

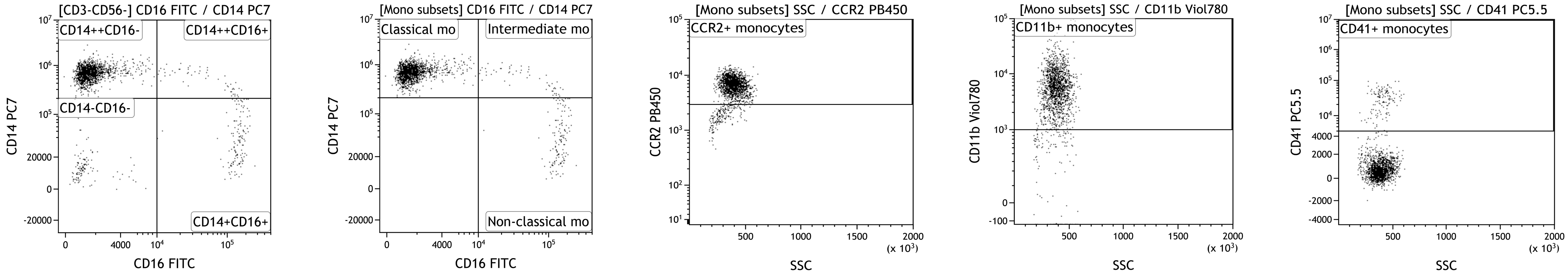

Gate	Number	%Gated
All	1.842	100,00
CD14-CD16-	117	6,35
CD14++CD16-	1.545	83,88
CD14++CD16+	53	2,88
CD14+CD16+	127	6,89

Gate	Number	%Gated
All	1.725	100,00
Classical mo	1.545	89,57
Intermediate mo	53	3,07
J--	0	0,00
Non-classical mo	127	7,36

Gate	Number	%Gated
All	1.725	100,00
CCR2+ monocytes	1.541	89,33
**Gate**	**Y-GMean**	
All	5.644,22	
CCR2+ monocytes	6.481,42	

Gate	Number	%Gated
All	1.725	100,00
CD11b+ monocytes	1.595	92,46
**Gate**	**Y-GMean**	
All	4.260,12	
CD11b+ monocytes	5.536,82	

Gate	Number	%Gated
All	1.725	100,00
CD41+ monocytes	131	7,59
**Gate**	**Y-GMean**	
All	1.147,16	
CD41+ monocytes	24.909,29	
